# A Mitochondrial Encoded Messenger at the Nucleus

**DOI:** 10.3390/cells7080105

**Published:** 2018-08-13

**Authors:** Cheryl Qian Ying Yong, Bor Luen Tang

**Affiliations:** 1 Department of Biochemistry, Yong Loo Lin School of Medicine, Singapore 117597, Singapore; a0127148@u.nus.edu; 2 NUS Graduate School for Integrative Sciences and Engineering, National University of Singapore, Singapore 117456, Singapore

**Keywords:** AMP-activated protein kinase (AMPK), humanin, mitochondria, mitochondrial open reading frame of the 12S rRNA-c (MOTS-c), mitochondrial unfolded protein response (UPR^mt^)

## Abstract

Mitochondria–nucleus (mitonuclear) retrograde signaling via nuclear import of otherwise mitochondrial targeted factors occurs during mitochondrial unfolded protein response (UPR^mt^), a mechanism that counters mitochondrial and cellular stresses. Other than nuclear encoded proteins, mitochondrial DNA (mtDNA)-encoded peptides, such as humanin, are known to have important pro-survival and metabolic regulatory functions. A recent report has indicated that another mtDNA-encoded peptide, the mitochondrial open reading frame of the 12S rRNA-c (MOTS-c), could translocate into the nucleus upon stress induction. In the nucleus, MOTS-c binds to DNA and regulates the transcription of stress response genes in concert with other transcription factors. This is the first clear example of a mitochondria-derived peptide (MDP) acting in the nucleus to affect transcriptional responses to stress. Thus, MOTS-c may bear some characteristics of a ‘mitokine’ factor that mediates mitohormesis, influencing cell survival as well as organismal health and longevity.

## 1. Introduction

The mitochondrion is often viewed as a reductive endosymbiont. Its biogenesis, maintenance and function are highly dependent on the nucleus. The composition of a mature mitochondrion is largely dependent on the import of nuclear encoded, cytoplasmically synthesized proteins. Mitochondrial functions are mainly regulated by the nucleus through anterograde nuclear–mitochondrial signaling. However, the mitochondrion, being critical for the regulation of cellular survival and metabolism, also feeds back to the nucleus during situations of stress that perturb mitochondrial function. The process of mitochondrial unfolded protein response (UPR^mt^) [1,2], analogous to the UPR occurring in the endoplasmic reticulum, initiates adaptive response during cellular stress. UPR^mt^ involves mitochondria—nucleus (mitonuclear) retrograde signaling, a process in which underlying signaling mechanisms are gradually becoming clearer in recent years [3]. 

**Figure 1:**
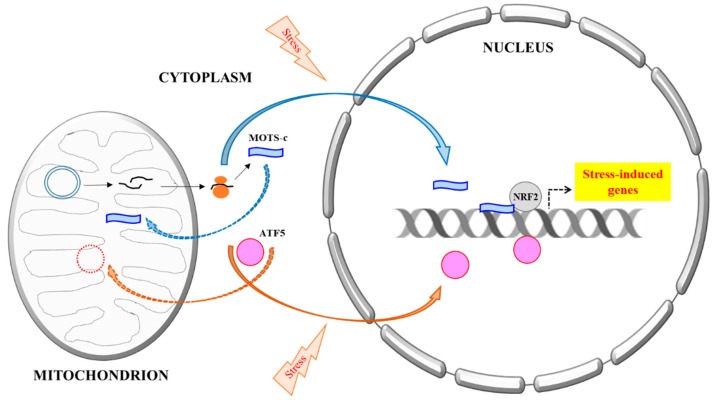
A schematic summary of the mitochondria DNA (mtDNA) encoded MOTS-c’s stress-induced nuclear translocation. All proteins known thus far that are primarily mitochondrial targeted, but could also be translocated into the nucleus, are all encoded by nuclear DNA. A prime example is ATF5, a mediator of Mitochondrial unfolded protein response (UPR^mt^). Activating Transcription Factor 5 (ATF5) is normally targeted and imported to the mitochondria, but could become nuclear localized during mitochondrial stress and mitochondrial import impairment, where it regulates transcriptional response to the stress. MOTS-c, however, is mtDNA encoded. It is likely synthesized in the cytoplasm and could eventually be localize to either mitochondria or the nucleus. Stress increases MOTS-c nuclear translocation in a manner that is dependent on AMPK activation, but the mechanism is not yet clear. In the nucleus, MOTS-c binds to DNA, particularly the promoters of stress-induced genes, as well as transcription factors, such as nuclear factor erythroid 2-related factor 2 (NRF2), and modulates transcriptional response to stress.

UPR^mt^ could be triggered by a range of mitochondrial perturbations, including excessive protein misfolding [1], inhibition of mitochondrial transcription and translation [4], as well as impairment of the electron transport chain (ETC) activity and elevation of reactive oxygen species (ROS) [5]. Although UPR^mt^ is a complex process, a relatively well-understood mechanism of mitonuclear signaling that occurs is a change in mitochondrial import of Activating Transcription Factor associated with Stress 1 (ATFS-1), first delineated in *C. elegans* [6], or its mammalian orthologue Activating Transcription Factor 5 (ATF5) [7]. Under basal conditions, the N-terminal mitochondrial targeting signal efficiently mediates ATFS-1/ATF5 import into mitochondria. When mitochondrial import becomes less efficient with mitochondrial stress, their C-terminal nuclear localization signal (NLS) will facilitate nuclear import of accumulating cytoplasmic ATFS-1/ATF5 instead (see Figure 1). In the nucleus, these factors could promote the transcription of genes encoding components of mitochondrial import, proteostasis promoting chaperone proteins, as well as anti-oxidative enzymes that will restore mitochondrial dysfunction. However, it should be noted that in mammalian cells, regulation of UPR^mt^ is likely to be more complex and involves other factors. For example, recent evidence has suggested that several mitochondrial stressors elicit an integrated stress response that is mediated by another member of the ATF family, ATF4, instead of ATF5 [8].

UPR^mt^ has received a great deal of attention in recent years, as it has been proposed to be involved in a phenomenon known as mitohormesis, in which mild oxidative stress of mitochondrial origin promotes organismal health and longevity [5,9,10,11,12]. In fact, works in *C. elegans* have shown that a mitochondrial signal(s), stemming from mild oxidative stress due to increased ROS production from the ETC defects, could trigger mitochondrial stress responses even in a distal tissue [5]. Such a cell-non-autonomous signaling by a ‘mitokine’ could be mediated by Wnt has been indicated by a recent report [13]. The identity of a strictly mitochondrial-derived factor that could act as a mitokine is not yet known. However, the mitochondria do produce peptides, and one of these has now been shown to mediate transcriptional stress response by its translocation into the nucleus and interaction with DNA [14].

## 2. Mitochondria-Derived Peptides

The human mitochondrial DNA (mtDNA) encodes a total of 37 classically known genes, including 2 rRNAs, 22 tRNAs and 13 polypeptide subunits of the ETC complexes (except Complex II). Recent work has revealed that the rRNA loci contain small open reading frames (ORFs) that could be transcribed and translated into short peptides, termed mitochondria-derived peptides (MDPs) [15], which have biological activity.

The first MDP discovered is humanin, which was identified by three different groups based on its protective effect of neural cells against the toxicity of amyloid-β peptide [16], an anti-apoptotic protein that binds Insulin-like growth factor-binding protein-3 (IGFBP-3) [17] or Bax [18]. Humanin, as its rat orthologue rattin [19], primarily exhibits cytoprotective and anti-apoptotic effects. The peptide has been shown to be protective in the neurons against neurotoxic and ischemic injuries [16,19,20,21], in myocardial ischemia and reperfusion injury [22,23] and oxidative stress-induced changes in retinal pigment epithelial cells [24]. Humanin also has metabolic effects, as a potent humanin analog has been shown to increase glucose-stimulated insulin secretion [25] and hepatic triglyceride secretion [26]. More than conferring protection against mitochondrial dysfunction [24], humanin also promotes mitochondrial biogenesis through the elevation of a master transcriptional regulator Peroxisome proliferator-activated receptor gamma coactivator 1-α (PGC-1α) [27].

Humanin and rattin are known to be transcribed and translated as short ORFs from the 16S rRNA of mtDNA [18,28]. Depending on whether translation occurs in the mitochondria or cytosol (which uses slightly different genetic codes), either a 21-amino acid (aa) or a 24-aa peptide is produced. However, nuclear DNA also contains several highly homologous humanin-like ORF harboring sites that could potentially give rise to multiple humanin-like peptides [29]. A pseudo signal sequence could facilitate its secretion, and its pro-survival property has been attributed to its engagement and signaling through two cellular receptors. The first is the G protein-coupled formylpeptide receptor-like-1 (FPRL1), for which it may potentially compete with the neurotoxic amyloid-β [30]. Another is a receptor complex of the ciliary neurotrophic factor receptor alpha (CNTFR)-gp130 and the IL-27 receptor subunit, WSX-1 [20,31]. The latter tripartite receptor complex is linked to the JAK-STAT, PI3 kinase-AKT and MAP kinase pathways. More recently, a series of small humanin-like peptides (SHLPs) 1-6, have been identified by in silico ORF searches at the 16S rRNA locus [32]. The SHLPs, particularly SHLP2 and SHLP3, have activities that overlap with that of humanin [32,33].

The 12S rRNA locus of mtDNA also harbors a small ORF encoding mitochondrial open reading frame of the 12S rRNA-c (MOTS-c) [34,35]. MOTS-c major activity appears to be that of metabolic homeostasis in fats and muscles, acting at least partly via the activation of AMP-activated protein kinase (AMPK) [36]. However, a recent report has now indicated that MOTS-c is induced by stress to regulate the transcription of stress response genes through the translocation into the nucleus [14]. These findings are discussed further below. A summary of MDPs discovered to date is provided in Table 1.

## 3. MOTS-c’s Mitochondrial–Nuclear Translocation and Activity

Lee and colleagues first identified MOTS-c as a 16-aa peptide encoded within the 12S rRNA locus of mtDNA of human cells [34]. The 16-aa peptide is predicted to be cytoplasmically translated since the use of mitochondria-specific genetic code would give rise to stop codons. Analysis of expression profile changes after the treatment of human HEK293 cells with MOTS-c indicated an altered expression of enzymes involved in the folate-methionine cycle and purine synthesis *de novo*. These changes resulted in the accumulation of 5-aminoimidazole-4-carboxamide ribonucleotide (AICAR), an activator of AMPK. Not surprisingly, cells over-expressing MOTS-c have altered glucose and fatty acid metabolism, and MOTS-c could target mouse skeletal muscle to increase AMPK activation [34,37], the expression of the insulin-responsive Glucose transporter 4 (GLUT4), as well as the promotion of insulin sensitivity. Most interestingly, MOTS-c treatment of mice alleviated a high fat diet-induced obesity and insulin resistance, acting in a manner that is, at least partially, pharmacologically analogous to the classical diabetes drug metformin [39]. In this regard, it is also notable that another recent report has found that circulating MOTS-c levels are lower in obese male children and adolescents compared to age-matched controls, and that its level is associated with insulin resistance [40]. 

In a more recent report, Lee and colleagues showed that the mitochondria-encoded MOTS-c could translocate into the nucleus and act as a transcription factor [14]. The authors detected low levels of endogenous MOTS-c in the nucleus under resting conditions in HEK293 cells. Likewise, a fraction of exogenously added or genetically over-expressed MOTS-c could be localized at the nucleus. Nuclear entry is not dependent on a classical NLS, which MOTS-c lacks, but could be abolished by mutating its hydrophobic core residues, suggesting that specific interactions with other factors may be required for nuclear translocation. Interestingly, metabolic stresses, such as glucose restriction, serum deprivation and oxidative stress, rapidly promote MOTS-c nuclear translocation. A common underlying stress metabolite that affects the translocation could be ROS, since N-acetylcysteine treatment inhibits the nuclear translocation. MOTS-c treatment activates AMPK, and its nuclear translocation appears to be also reciprocal dependent on AMPK activation, as both AMPK activators AICAR and metformin could promote MOTS-c nuclear translocation without eliciting any ROS production.

What role does MOTS-c play in the nucleus? MOTS-c binds to DNA and could be readily detected in chromatin extracts. As a key oxidative stress response transcription factor, nuclear factor erythroid 2-related factor 2 (NRF2) [41], is also known to functionally intersect with AMPK [42]; the authors therefore checked if there is an association between MOTS-c and NRF2. Indeed, an association between the two molecules could be demonstrated by co-immunoprecipitation using nuclear extracts. NRF2 and MOTS-c are transported independently into the nucleus, but they appear to associate within the nucleus. Importantly, electrophoretic mobility shift assays indicate that MOTS-c could bind directly to the antioxidant response element (ARE) sequences found in the promoter regions of NRF2 target genes. Chromatin immunoprecipitation followed by quantitative PCR (ChIP-qPCR) shows that this binding to the promoter regions of NRF2-reponsive genes, such as *HO-1* and *NQO1*, increases with stress, which also corresponds to an increase in NRF2 binding. By interacting with both cis elements and trans factors, MOTS-c could thus modulate DNA binding of transcription factors, such as NRF2, to promoter regions. In fact, a portion of genes up-regulated by MOTS-c over-expression are NRF2 target genes, which include two ATF family members, ATF1 and ATF7. MOTS-c co-immunoprecipitates with ATF1 in a stress-enhanced manner. Indeed, wild-type and nuclear translocation competent MOTS-c expression in cells makes them more resistant against metabolic stress, and this property is lost in MOTS-c mutants that could no longer bind to DNA. Therefore, mitochondrial MOTS-c could mediate cellular responses to metabolic stress by regulating nuclear gene transcription in conjunction with transcription factors that regulate stress response. 

## 4. New Perspectives

There are a number of proteins that are known to be dual targeted to both the mitochondria and the nucleus [43,44]. These include key stress and survival mediators, such as Tp53 [45] and the Forkhead box O3a (FOXO3a) [46]. In recent years, several moonlighting functions for primarily mitochondria-targeted proteins in the nucleus have also been demonstrated [44]. The tricarboxylic acid cycle enzyme, fumarase, could be recruited to the nucleus upon DNA damage induction and has an apparent role in DNA repair [47]. The mitochondrial pyruvate dehydrogenase complex (PDC) has been shown to have a presence in the nucleus, where it may act as a transcript co-activator [48] or facilitate the generation of acetyl-CoA in situ for histone acetylases [49]. In *C. elegans*, a nuclear form of the mitochondrial monooxygenase CLK-1 is known to be functional in regulating mitochondrial stress response [50]. However, all these proteins are derived from nuclear DNA-encoded transcripts. MOTS-c appears to be the first identified mtDNA-encoded nuclear transcription modulator.

MOTS-c is apparently synthesized in the cytoplasm and could be selectively targeted to the nucleus upon stress induction (see Figure 1). This intriguing finding by Lee and colleagues [14] has surfaced a number of equally interesting questions. The first pertains to its function. Although cytoplasmically translated MOTS-c could be eventually localized to the mitochondria, its actual function within the mitochondria is unclear. This is likewise the case for humanin and the SHLPs. One possibility could be that MOTS-c regulates the transcription of mtDNA-encoded genes, thereby playing a role in mitochondrial function. Another possibility, which is suggested by Lee and colleagues, is that the MDPs are relics of the co-evolutionary history of the ancestral cells participating in eukaryogenesis [51]. Therefore, the MDPs act primarily as means for directional communication between the mitochondria and the nucleus, similar to the way Gram-positive bacteria communicate with each other during quorum sensing [52]. This communications would be necessary as the endosymbionts adjust and adapt to each other’s metabolic needs and outputs. It is perhaps unsurprising that some role in metabolic regulation is a common feature of the MDPs. As the endosymbiotic relationship evolves, some of these molecules may become more specifically adapted for retrograde mitonuclear stress signaling. 

On the other hand, how MOTS-c exerts its metabolic effect, particularly when administered systemically, is also unclear. No specific signal receptor has been identified for MOTS-c yet, and AMPK is the only molecule it is known to functionally interact with. However, MOTS-c’s implicated function as a transcriptional regulator now provides a whole new perspective on its mode of action. In this regard, MOTS-c is a small peptide which could potentially diffuse freely through the openings of the nuclear pores. Its selective accumulation or retention in the nucleus could be facilitated by its interaction with the chromatin, and a specific facilitated import system would appear to be unnecessary. However, MOTS-c’s selective and AMPK-dependent nuclear translocation indicates a much more complex and regulated mode of nuclear import. Lee and colleagues’ data suggest that MOTS-c is not likely to be hitch-hiking on NRF2 to enter the nucleus. This, of course, does not rule out translocation mediated by other NLS-containing nuclear factors. The mechanisms underlying MOTS-c’s regulated nuclear translocation and the connection with AMPK would be an interesting pursuit for the near future. 

Perhaps more interestingly, MOTS-c could also be secreted from the cell-like humanin. As mentioned above, plasma MOTS-c levels appear to correlate with metabolic status, such as obesity and insulin resistance [39]. UPR^mt^ and mitonuclear retrograde stress signaling, particularly those arising from mild elevation in oxidative stress, have received much attention in recent years, as these have been proposed in multiple settings to be associated with organismal health, aging and longevity [4,5,9,10,11,12,53,54,55]. A mitochondrial stress signal, or a ‘mitokine’, could confer protection and promote survival, while priming the cell’s readiness for subsequent insults with increasing severity. Ristow has previously coined the term ‘mitohormesis’ for such a phenomenon [9]. Apparently, the range of action for this elusive mitokine is not limited within a cell, and at least in *C. elegans*, it has been shown that mitochondrial stress in one tissue type confers protection in other tissues [5]. In fact, a very recent report from Dillin’s laboratory has shown that retromer-dependent Wnt signaling is critical for this propagation of mitochondrial stress signals from the nervous system to peripheral tissues [13]. Wnt may thus be a prime candidate for a mitokine. The mitohormetic signal(s) are known to affect cellular and organismal aging pathways, which involve key factors that impinge on cell/tissue survival and metabolic health, such as the mechanistic target of rapamycin (mTOR) [56] and the nicotinamide adenine dinucleotide (NAD^+^)-SIRT1 pathways [10]. Activation of these pathways results in transcriptomic and epigenetic changes in the genome that promotes health and life span extension in model organisms [54,55]. MOTS-c also appears to bear some characteristics of a mitokine molecule. In this regard, it is worth noting that a MOTS-c m.1382A>C polymorphism, which is specific for the Northeast Asian population, may speculatively underlie the longer life span of the Japanese [38]. MOTS-c and other MDPs would no doubt be subjects of further investigation along this line of translational interest. 

## Figures and Tables

**Table 1:** A summary of mitochondria-derived peptides discovered to date.

Name	Origin and Properties	Activities
Humanin	21-aa (mitochondrial) or 24-aa (cytosol) peptide from open reading frame of mitochondrial 16S rRNA locus [16,17,18,28]. Also multiple humanin-like sequences from nuclear loci [29].	Cytoprotection against apoptotic agents, injuries and oxidative stress [16,17,18] Neuroprotection and cardioprotection [20,21,22,23,24] Insulin sensitivity [25] Mitochondrial biogenesis [27]
Rattin	38-aa rat orthologue of human humanin [19]	Cytoprotection against apoptotic agents and injuries [19]
Mitochondrial open reading frame of the 12S rRNA-c (MOTS-c)	16-aa peptide from open reading frame of mitochondrial 12S rRNA locus [34]	Mitochondrial metabolic regulation [34] Prevents high fat diet-induced obesity and insulin resistance in mice [34] Inhibits receptor activator of nuclear factor-κB ligand (RANKL)-induced osteoclast formation [37] m.1382A>C polymorphism correlation with longevity [38]
Small humanin-like peptide 1–6 (SHLP1–6)	20–38-aa peptide from open reading frame of mitochondrial 16S rRNA locus [32]	Mitochondrial metabolic regulation (SHLP2 and SHLP3) [32] Inhibition of apoptosis (SHLP2 and SHLP3) [32] Cell proliferation (SHLP2 and SHLP4) [32] Promotion of apoptosis (SHLP6) [32]

## References

[1] Zhao Q., Wang J., Levichkin I.V., Stasinopoulos S., Ryan M.T., Hoogenraad N.J. (2002). A mitochondrial specific stress response in mammalian cells. EMBO J..

[2] Fiorese C.J., Haynes C.M. (2017). Integrating the UPR^mt^ into the mitochondrial maintenance network. Crit. Rev. Biochem. Mol. Biol..

[3] Quirós P.M., Mottis A., Auwerx J. (2016). Mitonuclear communication in homeostasis and stress. Nat. Rev. Mol. Cell Biol..

[4] Houtkooper R.H., Mouchiroud L., Ryu D., Moullan N., Katsyuba E., Knott G., Williams R.W., Auwerx J. (2013). Mitonuclear protein imbalance as a conserved longevity mechanism. Nature.

[5] Durieux J., Wolff S., Dillin A. (2011). The cell-non-autonomous nature of electron transport chain-mediated longevity. Cell.

[6] Nargund A.M., Pellegrino M.W., Fiorese C.J., Baker B.M., Haynes C.M. (2012). Mitochondrial import efficiency of ATFS-1 regulates mitochondrial UPR activation. Science.

[7] Fiorese C.J., Schulz A.M., Lin Y.F., Rosin N., Pellegrino M.W., Haynes C.M. (2016). The transcription factor ATF5 mediates a mammalian mitochondrial UPR. Curr. Biol..

[8] Quirós P.M., Prado M.A., Zamboni N., D’Amico D., Williams R.W., Finley D., Gygi S.P., Auwerx J. (2017). Multi-omics analysis identifies ATF4 as a key regulator of the mitochondrial stress response in mammals. J. Cell Biol..

[9] Ristow M., Zarse K. (2010). How increased oxidative stress promotes longevity and metabolic health: The concept of mitochondrial hormesis (mitohormesis). Exp. Gerontol..

[10] Mouchiroud L., Houtkooper R.H., Moullan N., Katsyuba E., Ryu D., Cantó C., Mottis A., Jo Y.S., Viswanathan M., Schoonjans K. (2013). The NAD(+)/Sirtuin pathway modulates longevity through activation of mitochondrial UPR and FOXO signaling. Cell.

[11] Long Y.C., Tan T.M.C., Takao I., Tang B.L. (2014). The biochemistry and cell biology of aging: Metabolic regulation through mitochondrial signaling. Am. J. Physiol. Endocrinol. Metab..

[12] Yun J., Finkel T. (2014). Mitohormesis. Cell Metab..

[13] Zhang Q., Wu X., Chen P., Liu L., Xin N., Tian Y., Dillin A. (2018). The mitochondrial unfolded protein response is mediated cell-non-autonomously by retromer-dependent Wnt signaling. Cell.

[14] Kim K.H., Son J.M., Benayoun B.A., Lee C. (2018). The mitochondrial-encoded peptide MOTS-c translocates to the nucleus to regulate nuclear gene expression in response to metabolic stress. Cell Metab..

[15] Kim S.J., Xiao J., Wan J., Cohen P., Yen K. (2017). Mitochondrially derived peptides as novel regulators of metabolism. J. Physiol..

[16] Hashimoto Y., Niikura T., Ito Y., Sudo H., Hata M., Arakawa E., Abe Y., Kita Y., Nishimoto I. (2001). Detailed characterization of neuroprotection by a rescue factor humanin against various Alzheimer’s disease-relevant insults. J. Neurosci..

[17] Ikonen M., Liu B., Hashimoto Y., Ma L., Lee K.W., Niikura T., Nishimoto I., Cohen P. (2003). Interaction between the Alzheimer’s survival peptide humanin and insulin-like growth factor-binding protein 3 regulates cell survival and apoptosis. Proc. Natl. Acad. Sci. USA.

[18] Guo B., Zhai D., Cabezas E., Welsh K., Nouraini S., Satterthwait A.C., Reed J.C. (2003). Humanin peptide suppresses apoptosis by interfering with Bax activation. Nature.

[19] Caricasole A., Bruno V., Cappuccio I., Melchiorri D., Copani A., Nicoletti F. (2002). A novel rat gene encoding a Humanin-like peptide endowed with broad neuroprotective activity. FASEB J..

[20] Hashimoto Y., Kurita M., Aiso S., Nishimoto I., Matsuoka M. (2009). Humanin inhibits neuronal cell death by interacting with a cytokine receptor complex or complexes involving CNTF receptor alpha/WSX-1/gp130. Mol. Biol. Cell.

[21] Zhao S.T., Huang X.T., Zhang C., Ke Y. (2012). Humanin protects cortical neurons from ischemia and reperfusion injury by the increased activity of superoxide dismutase. Neurochem. Res..

[22] Muzumdar R.H., Huffman D.M., Calvert J.W., Jha S., Weinberg Y., Cui L., Nemkal A., Atzmon G., Klein L., Gundewar S. (2010). Acute humanin therapy attenuates myocardial ischemia and reperfusion injury in mice. Arterioscler. Thromb. Vasc. Biol..

[23] Thummasorn S., Apaijai N., Kerdphoo S., Shinlapawittayatorn K., Chattipakorn S.C., Chattipakorn N. (2016). Humanin exerts cardioprotection against cardiac ischemia/reperfusion injury through attenuation of mitochondrial dysfunction. Cardiovasc. Ther..

[24] Sreekumar P.G., Ishikawa K., Spee C., Mehta H.H., Wan J., Yen K., Cohen P., Kannan R., Hinton D.R. (2016). The mitochondrial-derived peptide Humanin protects RPE cells from oxidative stress, senescence, and mitochondrial dysfunction. Investig. Ophthalmol. Vis. Sci..

[25] Kuliawat R., Klein L., Gong Z., Nicoletta-Gentile M., Nemkal A., Cui L., Bastie C., Su K., Huffman D., Surana M. (2013). Potent humanin analog increases glucose-stimulated insulin secretion through enhanced metabolism in the β cell. FASEB J..

[26] Gong Z., Su K., Cui L., Tas E., Zhang T., Dong H.H., Yakar S., Muzumdar R.H. (2015). Central effects of humanin on hepatic triglyceride secretion. Am. J. Physiol. Endocrinol. Metab..

[27] Qin Q., Jin J., He F., Zheng Y., Li T., Zhang Y., He J. (2018). Humanin promotes mitochondrial biogenesis in pancreatic MIN6 β-cells. Biochem. Biophys. Res. Commun..

[28] Maximov V., Martynenko A., Hunsmann G., Tarantul V. (2002). Mitochondrial 16S rRNA gene encodes a functional peptide, a potential drug for Alzheimer’s disease and target for cancer therapy. Med. Hypotheses.

[29] Bodzioch M., Lapicka-Bodzioch K., Zapala B., Kamysz W., Kiec-Wilk B., Dembinska-Kiec A. (2009). Evidence for potential functionality of nuclearly-encoded humanin isoforms. Genomics.

[30] Ying G., Iribarren P., Zhou Y., Gong W., Zhang N., Yu Z.X., Le Y., Cui Y., Wang J.M. (2004). Humanin, a newly identified neuroprotective factor, uses the G protein-coupled formylpeptide receptor-like-1 as a functional receptor. J. Immunol..

[31] Kim S.J., Guerrero N., Wassef G., Xiao J., Mehta H.H., Cohen P., Yen K. (2016). The mitochondrial-derived peptide humanin activates the ERK1/2, AKT, and STAT3 signaling pathways and has age-dependent signaling differences in the hippocampus. Oncotarget.

[32] Cobb L.J., Lee C., Xiao J., Yen K., Wong R.G., Nakamura H.K., Mehta H.H., Gao Q., Ashur C., Huffman D.M. (2016). Naturally occurring mitochondrial-derived peptides are age-dependent regulators of apoptosis, insulin sensitivity, and inflammatory markers. Aging.

[33] Okada A.K., Teranishi K., Lobo F., Isas J.M., Xiao J., Yen K., Cohen P., Langen R. (2017). The mitochondrial-derived peptides, HumaninS14G and small Humanin-like peptide 2, exhibit chaperone-like activity. Sci. Rep..

[34] Lee C., Zeng J., Drew B.G., Sallam T., Martin-Montalvo A., Wan J., Kim S.J., Mehta H., Hevener A.L., de Cabo R. (2015). The mitochondrial-derived peptide MOTS-c promotes metabolic homeostasis and reduces obesity and insulin resistance. Cell Metab..

[35] Zarse K., Ristow M. (2015). A mitochondrially encoded hormone ameliorates obesity and insulin resistance. Cell Metab..

[36] Hardie D.G., Schaffer B.E., Brunet A. (2016). AMPK: An energy-sensing pathway with multiple inputs and outputs. Trends Cell Biol..

[37] Wei M., Gan L., Sha X., Lu H.Y., Jiang Y., Lei X.Y., Xu C., Ruan B.J., Wang L., Lu Z.F. (2016). Mitochondria related peptide MOTS-c suppresses ovariectomy-induced bone loss via AMPK activation. Biochem. Biophys. Res. Commun..

[38] Fuku N., Pareja-Galeano H., Zempo H., Alis R., Arai Y., Lucia A., Hirose N. (2015). The mitochondrial-derived peptide MOTS-c: A player in exceptional longevity?. Aging Cell.

[39] Foretz M., Guigas B., Bertrand L., Pollak M., Viollet B. (2014). Metformin: From mechanisms of action to therapies. Cell Metab..

[40] Du C., Zhang C., Wu W., Liang Y., Wang A., Wu S., Zhao Y., Hou L., Ning Q., Luo X. (2018). Circulating MOTS-c levels are decreased in obese male children and adolescents and associated with insulin resistance. Pediatr. Diabetes.

[41] Hayes J.D., Dinkova-Kostova A.T. (2014). The Nrf2 regulatory network provides an interface between redox and intermediary metabolism. Trends Biochem. Sci..

[42] Joo M.S., Kim W.D., Lee K.Y., Kim J.H., Koo J.H., Kim S.G. (2016). AMPK facilitates nuclear accumulation of Nrf2 by phosphorylating at serine 550. Mol. Cell. Biol..

[43] Czypiorski P., Altschmied J., Rabanter L.L., Goy C., Jakob S., Haendeler J. (2014). Outfielders playing in the infield: Functions of aging-associated “nuclear” proteins in the mitochondria. Curr. Mol. Med..

[44] Monaghan R.M., Whitmarsh A.J. (2015). Mitochondrial Proteins Moonlighting in the Nucleus. Trends Biochem. Sci..

[45] Vaseva A.V., Marchenko N.D., Ji K., Tsirka S.E., Holzmann S., Moll U.M. (2012). p53 opens the mitochondrial permeability transition pore to trigger necrosis. Cell.

[46] Celestini V., Tezil T., Russo L., Fasano C., Sanese P., Forte G., Peserico A., Lepore Signorile M., Longo G., De Rasmo D. (2018). Uncoupling FoxO3A mitochondrial and nuclear functions in cancer cells undergoing metabolic stress and chemotherapy. Cell Death Dis..

[47] Yogev O., Yogev O., Singer E., Shaulian E., Goldberg M., Fox T.D., Pines O. (2010). Fumarase: A mitochondrial metabolic enzyme and a cytosolic/nuclear component of the DNA damage response. PLoS Biol..

[48] Chueh F.Y., Leong K.F., Cronk R.J., Venkitachalam S., Pabich S., Yu C.L. (2011). Nuclear localization of pyruvate dehydrogenase complex-E2 (PDC-E2), a mitochondrial enzyme, and its role in signal transducer and activator of transcription 5 (STAT5)-dependent gene transcription. Cell. Signal..

[49] Sutendra G., Kinnaird A., Dromparis P., Paulin R., Stenson T.H., Haromy A., Hashimoto K., Zhang N., Flaim E., Michelakis E.D. (2014). A nuclear pyruvate dehydrogenase complex is important for the generation of acetyl-CoA and histone acetylation. Cell.

[50] Monaghan R.M., Barnes R.G., Fisher K., Andreou T., Rooney N., Poulin G.B., Whitmarsh A.J. (2015). A nuclear role for the respiratory enzyme CLK-1 in regulating mitochondrial stress responses and longevity. Nat. Cell Biol..

[51] Dacks J.B., Field M.C., Buick R., Eme L., Gribaldo S., Roger A.J., Brochier-Armanet C., Devos D.P. (2016). The changing view of eukaryogenesis—Fossils, cells, lineages and how they all come together. J. Cell Sci..

[52] Verbeke F., De Craemer S., Debunne N., Janssens Y., Wynendaele E., Van de Wiele C., De Spiegeleer B. (2017). Peptides as quorum sensing molecules: Measurement techniques and obtained levels in vitro and in vivo. Front. Neurosci..

[53] Owusu-Ansah E., Song W., Perrimon N. (2013). Muscle mitohormesis promotes longevity via systemic repression of insulin signaling. Cell.

[54] Tian Y., Garcia G., Bian Q., Steffen K.K., Joe L., Wolff S., Meyer B.J., Dillin A. (2016). Mitochondrial stress induces chromatin reorganization to promote longevity and UPR(mt). Cell.

[55] Merkwirth C., Jovaisaite V., Durieux J., Matilainen O., Jordan S.D., Quiros P.M., Steffen K.K., Williams E.G., Mouchiroud L., Tronnes S.U. (2016). Two conserved histone demethylases regulate mitochondrial stress-induced longevity. Cell.

[56] Wei Y., Zhang Y.J., Cai Y., Xu M.H. (2015). The role of mitochondria in mTOR-regulated longevity. Biol. Rev. Camb. Philos. Soc..

